# Porcine Model of the Arterial Switch Operation: Implications for Unique Strategies in the Management of Hypoplastic Left Ventricles

**DOI:** 10.1007/s00246-020-02507-8

**Published:** 2020-11-30

**Authors:** Anthony Azakie, John P. Carney, Matthew T. Lahti, Richard W. Bianco, Michelle J. Doyle, Rajat Kalra, Cindy M. Martin

**Affiliations:** 1 Experimental Surgical Services Laboratory, Department of Surgery, University of Minnesota Medical School, Minneapolis, MN 55455 USA; 2 Cardiovascular Division, Department of Medicine, University of Minnesota Medical School, Minneapolis, MN USA

**Keywords:** Porcine model, Arterial switch operation, Hypoplastic left heart syndrome, Left ventricle hypoplasia, Right ventricle failure, Heart failure

## Abstract

There are no reports on the performance of the arterial switch operation (ASO) in a normal heart with normally related great vessels. The objective of this study was to determine whether the ASO could be performed in a healthy animal model. Cardiopulmonary bypass (CPB) and coronary translocation techniques were used to perform ASO in neonatal piglets or a staged ASO with prior main pulmonary artery (PA) banding. Primary ASO was performed in four neonatal piglets. Coronary translocation was effective with angiograms confirming patency. Piglets could not be weaned from CPB due to right ventricle (RV) dysfunction. To improve RV function for the ASO, nine piglets had PA banding. All survived the procedure. Post-banding RV pressure increased from a mean of 20.3 ± 2.2 mmHg to 36.5 ± 7.3 mmHg (*p* = 0.007). At 58 ± 1 days post-banding, piglets underwent cardiac MRIs revealing RV hypertrophy, and RV pressure overload with mildly reduced RV function. Catheterization confirmed RV systolic pressures of 84.0 ± 6.7 mmHg with LV systolic pressure 83.3 ± 6.7 mmHg (*p* = 0.43). The remaining five PA banded piglets underwent ASO at 51 ± 0 days post-banding. Three of five were weaned from bypass with patent coronary arteries and adequate RV function. We were able to successfully perform an arterial switch with documented patent coronary arteries on standard anatomy great vessels in a healthy animal model. To our knowledge this is the first time this procedure has been successfully performed. The model may have implications for studying the failing systemic RV, and may support a novel approach for management of borderline, pulsatile left ventricles.

## Introduction

The arterial switch operation (ASO) has become the standard of care for D-transposition of the great arteries even in the setting of unusual coronary artery patterns [1–4]. The operation also has indications in the setting of L-transposed great vessels, as part of the double switch procedure. In both D and L transposed great vessels, the coronaries are usually facing a sinus to which they can be translocated.

There are no known reports on the performance of the arterial switch in a normal heart with normally related great vessels. In normal hearts with usual coronary artery patterns and normally related great vessels, the right coronary artery may be distant from the main PA root, which is anterior and to the left. Furthermore, translocation of the left coronary artery may result in hairpin looping of the left main to the left anterior descending coronary artery axis. Due to this difficulty in both human and animal models, experimental models of the arterial switch presented in the literature merely replicate the anastomoses required in an arterial switch without chronically switching the subpulmonic and systemic ventricles [5, 6].

In the current study, we report the successful completion of the arterial switch operation in a novel porcine model, in a normal heart. The study not only provides the first animal model of the ASO, it demonstrates that the coronary arteries in normally related great vessels are “switchable”. This first report of the ASO in a normal heart may provide a potentially novel approach to the management of variants of hypoplastic left heart syndrome (HLHS) where the left ventricle has borderline size or function. An arterial switch in a heart with non-apex forming, but pulsatile, left ventricle may be an initial step for palliation of HLHS variants.

## Methods

Approval was obtained from the laboratory’s Institutional Animal Care and Use Committee (IACUC) prior to the start of the experiment.

### Study Design

Animals undergoing arterial switch were divided into two study groups: (1) naïve and (2) pulmonary artery (PA) banded animals. In Group 1, (*n* = 4) the arterial switch was performed on healthy naïve animals, which had not been previously intervened upon. In Group 2a, (*n* = 4) piglets underwent pulmonary artery (PA) banding and 8 weeks post-banding underwent cardiac MRI and hemodynamic assessment. In Group 2b, (*n* = 5) piglets underwent PA banding and were recovered for a period of 7–8 weeks prior to the arterial switch operation.

### Animals

Yorkshire cross piglets aged 3–5 weeks, weighing 7–12 kg were used for this study. Animals were fed Harlan Teklad Swine Chow #8753. They were fed twice daily and allowed to consume their food throughout the day. All animals were given water ad libitum throughout the study.

### Fasting/Pre-operative Program

Animals were fasted for 12–24 h prior to anesthetic events with water provided ad lib. Sustained release (SR) Buprenorphine (SQ) was used for pre- and post- operative analgesia at a dose of 0.1–0.3 mg/kg 12–24 h prior to the surgical procedure.

### Induction

Animals were sedated with 1–8 mg/kg Telazol®/1–3 mg/kg xylazine IM. After allowing 5–10 min for the drug cocktail to take effect, the ear vein was aseptically prepped and a catheter placed. Anesthesia was induced using 2–6 mg/kg Propofol IV. Supportive fluids, 0.9% normal saline (NaCl), were administered through the catheter. Animals were endotracheally intubated for mechanical ventilation at 10–15 breaths per minute, 4 L O_2_/min, and 1–4% isoflurane. An antibiotic, 5 mg/kg ceftiofur IM, and corticosteroid, 250 mg methylprednisolone IV were administered prior to incision. The animal was positioned in the supine position, aseptically prepped, and draped. Heart rate, respiratory rate, oxygen saturation, body temperature and intravenous fluid infusion rate were monitored.

### Surgical Preparation

A cut-down incision was performed in the left or right inguinal area. The femoral artery and vein were catheterized with a modified Seldinger technique and connected to fluid-filled monitoring lines for measurement of central venous and arterial pressure throughout the case, and the administration of drugs.

A midline sternotomy was performed and the pericardium opened and retracted. Polypropylene purse strings were placed in the thoracic aorta and right atrium in preparation for cardiopulmonary bypass (CPB) cannulation. The animal was anticoagulated with 250 units/kg heparin, and CPB cannulas placed and secured. Cardiopulmonary bypass with cooling to 25 °C were initiated.

### Arterial Switch

A cross clamp was placed distally on the ascending aorta. Del Nido cardioplegia (20 ml/kg) was administered to the aortic root and coronary arteries. Following arrest, the aorta was transected above the level of the sino-tubular junction and coronary buttons dissected and mobilized as necessary from the aortic root. The main pulmonary artery (MPA) was transected and trap door incisions were made to create two ostia attachment sites for the coronary buttons. The left coronary orifice/ostia is very close to the aortic valve annulus in neonatal pigs, and sits leftward close to the left–right commissure of the aortic valve. The coronary ostia were sewn to the pulmonary artery attachment sites with continuous 7–0 polypropylene suture. The subpulmonic root (transected proximal aortic root to transected distal pulmonary arteries) was reconstructed with pericardial patch material. A Lecompte maneuver was performed to complete the neo-systemic root (transected proximal pulmonary artery root with attached coronary ostia to transected distal aorta) and the great vessels switched. Cardiopulmonary bypass was terminated and a right atriotomy performed. An extensive septectomy was created in the atrial septum and the heart decompressed. Of note, the atrial septum is small, posterior and inferior, and its excision is easily complicated by posterior cardiac perforation. The right atriotomy was closed with continuous 5–0 polypropylene suture and CPB initiated with core warming of the animal to 35 °C.

With the arterial switch and septectomy completed, an additional 125 mg methylprednisolone and 3 mg/kg ceftiofur were administered IV. Mechanical ventilation was reinstated and the animal weaned from bypass utilizing epinephrine, phenylephrine and/or dobutamine for hemodynamic support once core warming was accomplished. Angiography was performed using peripheral access via the femoral artery and vein. Diagnostic catheters were advanced to both the right and left sides of the heart under fluoroscopy. Isovue-300 (Iopamidol Injection 61%) was injected into the heart via diagnostic catheters to determine patency of the coronary arteries and mapping of blood flow patterns following the switch.

### Pulmonary Artery Banding

Animals were anesthetized and prepared for sterile surgery as previously described. Animals were placed in the right decubitus position and a left 4^th^-space thoracotomy was performed to access the heart. The pericardium was opened and a purse string placed in the MPA for introduction of a pressure catheter. The animal was heparinized prior to placement of the pressure catheter. An umbilical tape was looped around the MPA and tightened while pulmonary artery pressure monitored. The umbilical tape was secured in the cinched position after the peak systolic pressure in the right ventricle had increased 50–75%. Once the umbilical tape was secured, the pressure catheter was removed, heparin was reversed (protamine, IV, approximate ratio of 10 mg protamine:1000 IU of heparin).

Prior to closing the thoracotomy, a local nerve block was created using 1–2 mg/kg of both lidocaine and bupivacaine, given IM at the incision site. The lungs were inflated and the chest cavity irrigated with a warm saline antibiotic solution (1 g of ampicillin in 1 l of sterile saline).

A #20–#30 drainage tube was secured in the chest through the 6th intercostal space, exteriorized through the skin, secured in place with a purse string and connected to a water sealed vacuum drainage reservoir. The ribs were approximated with three size 1 braided polyester sutures. The muscle and skin layers were closed in standard fashion using 2–0 and 3–0 absorbable suture. Isoflurane was discontinued either during or following the closure of the thoracotomy.

The chest tube was removed when the animal was breathing normally and negative pressure in the intrapleural space. Once verified to be stable, the animal was moved to the post-operative care unit for recovery. Furosemide 40 mg twice daily was administered to assist with volume status optimization. 7–8 weeks following MPA banding, five animals were anesthetized for cardiac imaging and five animals were anesthetized for the arterial switch operation as described.

### Cardiac Magnetic Resonance (CMR) Imaging Protocol

All studies were performed with a Siemens 1.5 T Aera scanner (Siemens, Malvern, PA) with phased-array coil systems. The examination included localizers to assess cardiac position and a standard segmented steady-state free-precession cine sequence to assess cardiac volumes and function. The imaging parameters were as follows: typical repetition time of 3.0–3.5 ms, echo time of 1.2–1.5 ms, in-plane spatial resolution of 1.8 × 1.4 mm, and temporal resolution of 35–40 ms. Short-axis images were acquired with a slice thickness of 6 mm from the level of the mitral valve to the apex of the left ventricle. Long axis cines were also obtained in the four-chamber and three-chamber views. The cardiac magnetic resonance examination sequences were gated with electrocardiography. Following this, contrast-enhanced magnetic resonance angiography was performed to evaluate the pulmonary arterial tree. Another smaller contrast injection was delivered and timed to opacify the main pulmonary artery and the proximal branch pulmonary arteries using automatic contrast bolus detection.

### Cardiac Magnetic Resonance Imaging Analysis

CMR analyses were performed by an experienced CMR cardiologist using standard software (Precession by Heart Imaging Technologies, Durham, NC). Left and right ventricular end-diastolic and end-systolic volumes and ejection fractions were quantified by planimetry of the end-diastolic and end-systolic endocardial borders on the short-axis cine images.

### Statistics

Data are presented (mean ± SD) and analyzed using Microsoft Excel 2016. Paired *t*-tests were used to compare groups, with statistical significance defined as *p* < 0.05.

## Results

**Fig. 1:**
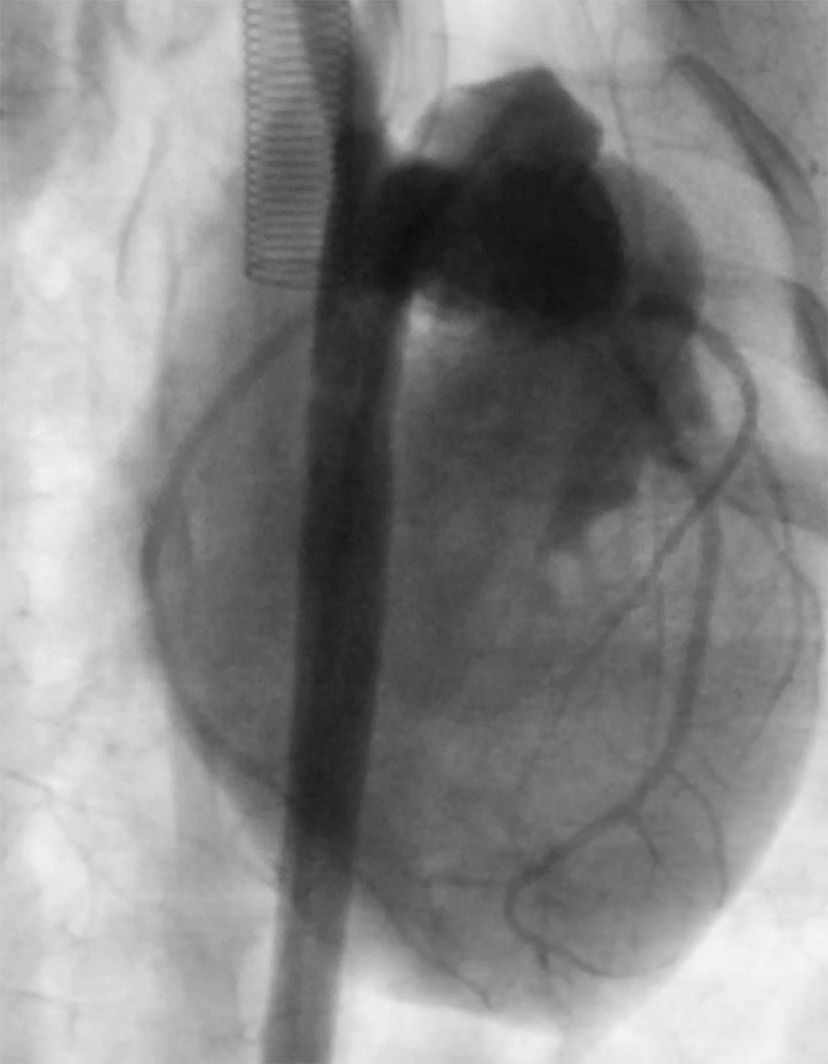
Angiography of translocated coronary arteries in 28-day-old piglet (Group 1) that underwent primary arterial switch operation with atrial septectomy. Both right and left coronary systems are widely patent

Primary arterial switch operation with atrial septectomy was performed in neonatal piglets (*n* = 4) in Group 1. The switch was successfully completed from a technical standpoint with documented patent coronary arteries on standard anatomy great vessels (Fig. 1). There were no instances of posterior cardiac perforation. However, weaning from cardiopulmonary bypass and short-term survival were complicated by significant RV dysfunction and hypoxia. Prior to termination, coronary angiography was performed to assess the patency of the vessels and outcomes of the coronary translocation. Both right and left coronaries were widely patent. In order to maintain RV conditioning and optimize ability to wean from bypass a PA banding protocol was developed.

**Fig. 2:**
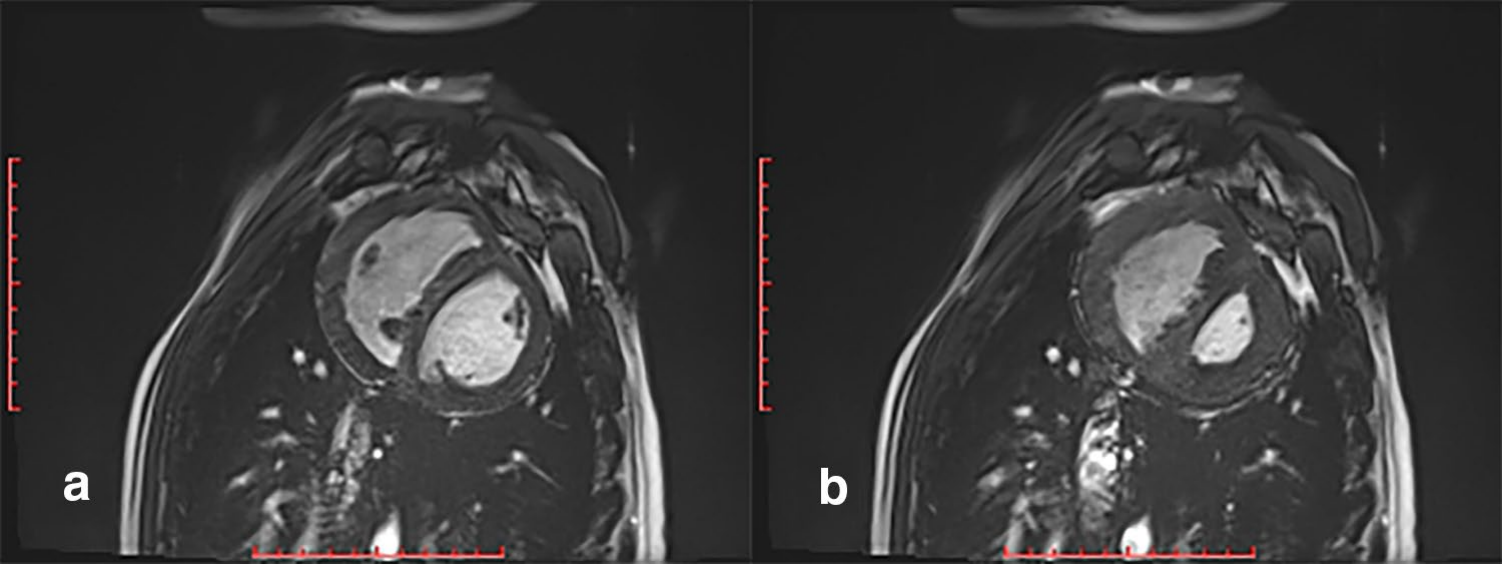
Cardiac MRI of 90-day-old piglet 57 days post-PA banding (Group 2a). Note the RV hypertrophy (**a** and **b**) and D-shaped left ventricular cavity (**b**) indicative of RV pressure overload

**Fig. 3:**
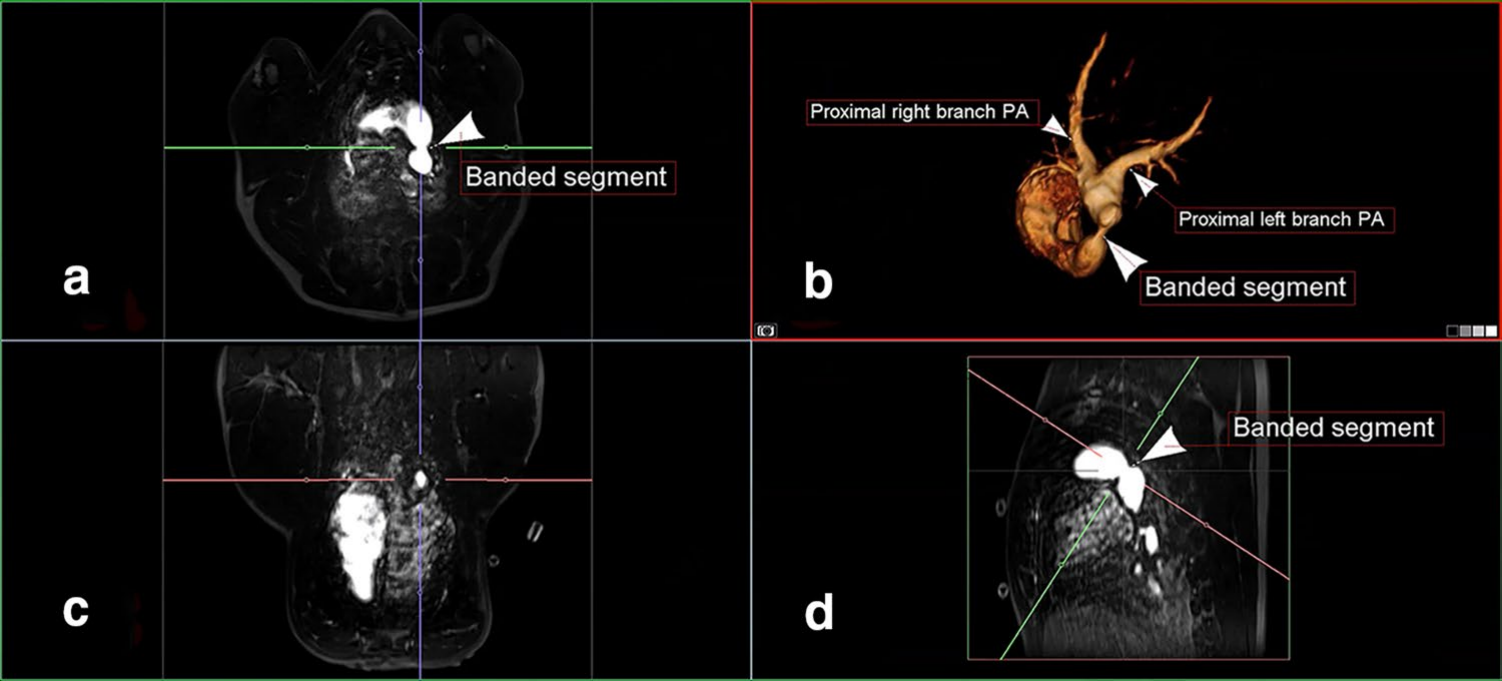
Contrasted-enhanced magnetic resonance angiogram of the pulmonary arterial tree. The banded segment of the main pulmonary artery is visualized in axial (**a**), three-dimensional (**b**), coronal (**c**), and sagittal (**d**) projections. *PA* pulmonary artery

Piglets in Group 2a (*n* = 4) had PA banding at an average age of 30.3 ± 1.5 days and weight of 5.8 ± 0.2 kg. RV pressure increased from 20.3 ± 2.2 to 36.5 ± 7.3 mmHg (*p* = 0.007) after placing the band. All survived the banding procedure. At 58 ± 1 days post-banding, piglets (*n* = 3) underwent cardiac MRIs revealing RV hypertrophy, evidence of RV pressure overload with mildly reduced RV function with RVEF 41 ± 3% and normal left ventricular function (LV) with LVEF 60 ± 2% (Fig. 2). A magnetic resonance angiogram of the portion of the main pulmonary artery that was banded is depicted in Fig. 3. Hemodynamic assessment was performed via intracardiac catheterization revealed that the peak systolic RV pressure increased from 36.5 ± 7.3 to 84.0 ± 6.7 mmHg (*p* = 0.0004) confirming that the RV pressures were equal to the LV peak systolic pressures averaging 83.3 ± 6.7 mmHg (*p* = 0.43). Cardiac hemodynamic data are presented in Table 1.

**Table 1:** Assessment of cardiac hemodynamics using intracardiac catheterization before and after pulmonary artery banding

Group	Pressure (mmHg)*				*p* Values*		
	Pre-banding RV peak	Post-banding RV peak	Term RV peak	Term LV peak	Pre-banding RV peak vs. post-banding RV peak	Post-banding RV peak vs. term RV peak	Term RV peak vs. term LV peak
2a (*n* = 4)	20.3 ± 2.2	36.5 ± 7.3	84.0 ± 6.7	83.3 ± 6.7	*p* = 0.007	*p* = 0.0004	*p* = 0.43
2b (*n* = 5)	21.6 ± 3.0	38.4 ± 7.5	NR	NR	*p* = 0.003	NA	NA

*NR* not recorded, *NA* not applicable

**Fig. 4:**
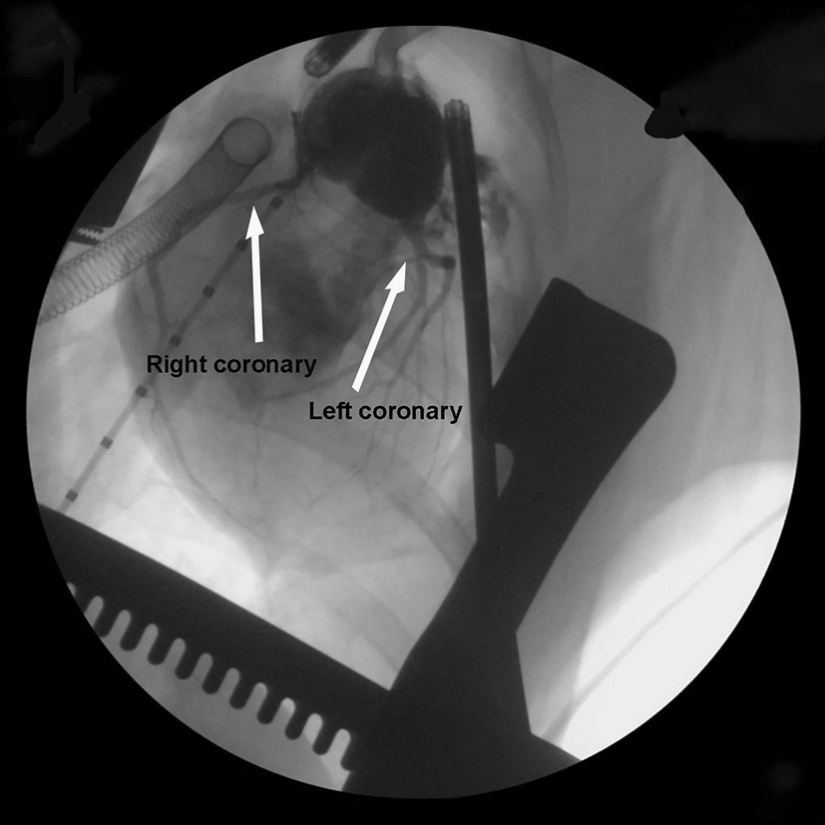
Angiogram of 82-day-old piglet (Group 2b) that had arterial switch operation with atrial septectomy. The animal was successfully weaned from cardiopulmonary bypass, with good RV function and the translocated coronaries are widely patent, indicated by white arrows. Prior PA banding was performed at 31 days of age

Piglets in Group 2b (*n* = 5) had PA banding at an average age of 31.8 ± 0.8 days and weight of 5.9 ± 0.2 kg. RV pressure increased from 21.6 ± 3.0 to 38.4 ± 7.5 mmHg (*p* = 0.003) after placing the band. All animals survived the banding procedure. At 51 ± 0 days post-banding piglets were prepared for the ASO. Piglets were 82.8 ± 0.8 days of age and 24.9 ± 2.8 kg at the time of the ASO. The arterial switch operation was completed in all five piglets. Three of the five piglets were successfully weaned from bypass for 35–55 min prior to termination utilizing epinephrine, phenylephrine and/or dobutamine for hemodynamic support. Of the three piglets weaned from CPB, the systolic arterial blood pressure was 68.0 ± 5.5 mmHg, diastolic blood pressure was 38.0 ± 4.9 mmHg, heart rate ranged from 98 to 138 bpm and hemoglobin O_2_ saturation ranged from 68 to 73%, presented in Table 2. Prior to termination, angiography was performed to evaluate coronary arterial anatomy (Fig. 4).

**Table 2:** Cardiac hemodynamics measured by intracardiac catheterization following arterial switch and weaning from cardiopulmonary bypass

Group	Arterial blood pressure (mmHg)		Heart rate (BPM)	O_2_ saturation (%)
	Systolic	Diastolic		
2b (*n* = 3)	68.0 ± 5.5	38.0 ± 4.9	98–138	68–73

**Fig. 5:**
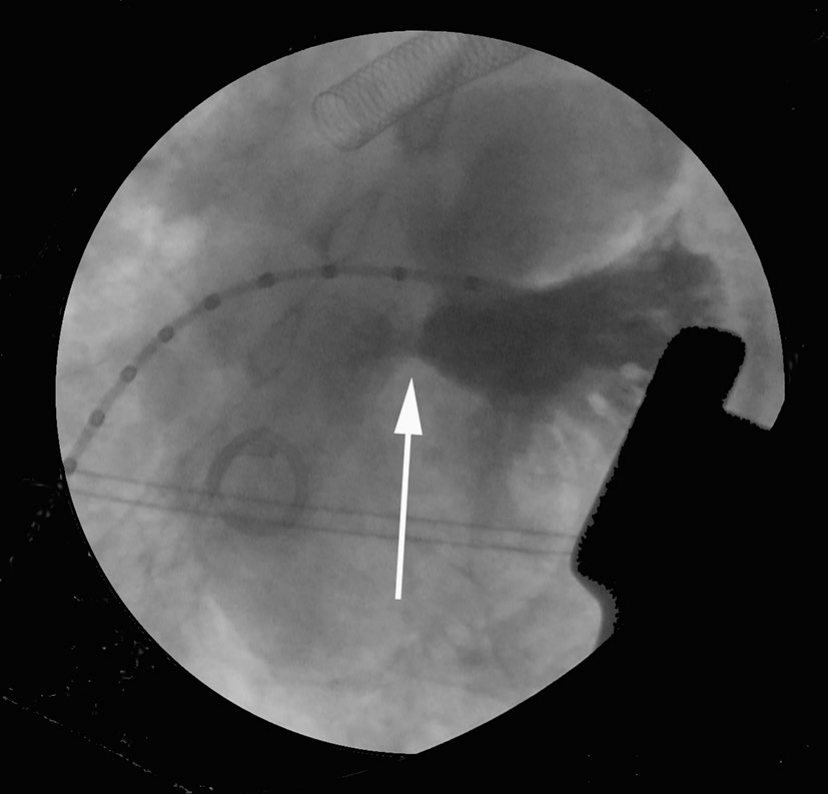
Left atrial contrast injection in after arterial switch in a piglet with prior PA banding. There is limited mixing into the RA with streaming of pulmonary venous blood to the LV/PA

**Fig. 6:**
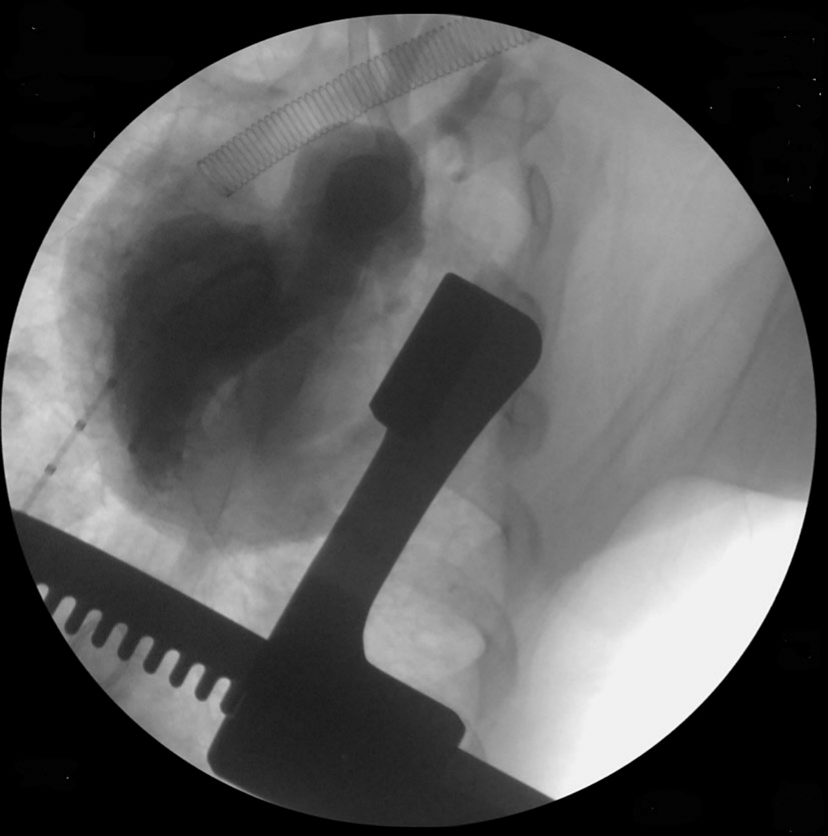
Right heart angiography following arterial switch in piglets with prior PA banding. Right atrial contrast streams to the aorta with little “washout” from the left atrium

The main complication from the procedure was hypoxemia due to creation of transposition physiology. There was limited mixing across the atrial defect. Contrast injection showed streaming of left atrial blood to the main PA and little mixing into the RA (Fig. 5) Injection into the right heart showed little if any washout from the LA, with streaming of RA blood to the aorta (Fig. 6).

**Fig. 7:**
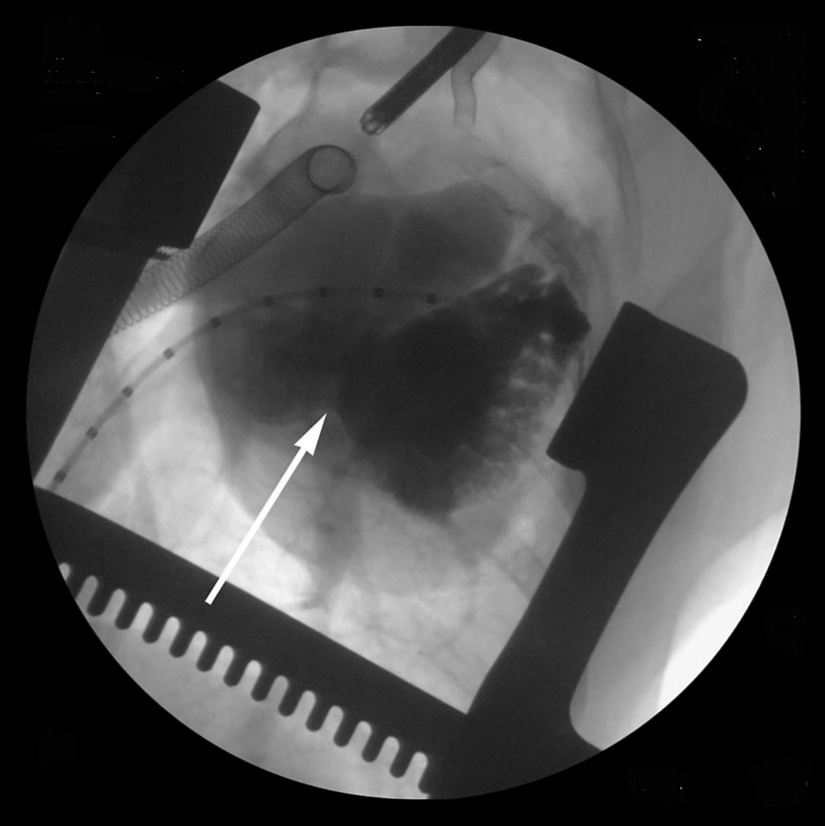
A more aggressive septectomy that extends inferiorly to the coronary sinus and IVC results in improved mixing after arterial switch in a piglet model with prior PA banding. Contrast fills both the left atrium and right atrium through the septectomy (white arrow) when injected into the left atrium

The animals displayed adequate RV function but still encountered significant issues with poor oxygenation. After thorough evaluation, the hypoxia was felt to be secondary to inadequate atrial level mixing. It was determined that performing a more radical atrial septectomy would be the best option to improve oxygenation through enhanced atrial level mixing. With this enhanced atrial septectomy the piglets demonstrated a significant improvement in atrial level mixing (Fig. 7). Piglets maintained adequate oxygenation and with slow weaning of cardiac bypass circuit and utilization of inotropic and vasoactive agents maintained adequate RV function.

## Discussion

### Creation of the Model

We first attempted to perform a double switch (arterial and atrial) operation in 4-week-old piglets with the intention of placing the RV in the systemic circulation with a septated heart to study the evolution of RV failure. Unfortunately during the initial procedures, we determined that given the shallow nature of the piglet atria and the acute angle at which the IVC enters the right atrium that an atrial switch would not be anatomically possible at this age. We opted to proceed with performing an arterial switch with a concomitant creation of an atrial septal defect via an atrial septectomy to allow adequate systemic oxygenation. This model would allow us to create a high pressure right ventricle and a low pressure left ventricle, but would necessitate some degree of hypoxia. We were able to successfully perform an arterial switch with documented patent coronary arteries on standard anatomy great vessels in a large animal model. To our knowledge this is the first time such a procedure has been successfully performed. Although the arterial switch and atrial septectomy were successful, the piglets could not be weaned from the cardiopulmonary bypass circuit due to right ventricular failure and issues with oxygenation. This was an anticipated complication. In an effort to allow sufficient RV function post arterial switch we performed PA banding 7–8 weeks prior to the arterial switch and atrial septectomy to allow RV “training” for high pressure needed to support systemic circulation post-ASO.

Piglets with banded PAs and a “trained RV” had successful arterial switch and atrial septectomy in the setting of a more complex surgical field due to significant adhesions from the prior PA banding surgery. The animals displayed adequate RV function but still encountered significant issues with inadequate atrial level mixing and hemoglobin O_2_ desaturation. We remained concerned that atrial mixing would continue to be an issue in the banded animals due to several factors. The prominent Eustachian valve/ridge and angle of the IVC to the atrial septum created a strong streaming current of deoxygenated blood from the IVC through the atrial septectomy to the left atrium. This limited the mixing of oxygenated blood returning to the left atrium with the right atrial blood. This impairment of mixing was exacerbated by the presence of significant RV hypertension causing ventricular stiffness and non-compliance, which further impaired left atrial to right atrial diastolic shunting. A more aggressive septectomy did allow for some improvement in left to right atrial diastolic shunting, and that, plus improved RV function of the post-PA-banded “trained” RV, allowed for animals to be weaned from cardiopulmonary bypass.

### Implications for HLHS and Borderline Left Ventricles

**Fig. 8:**
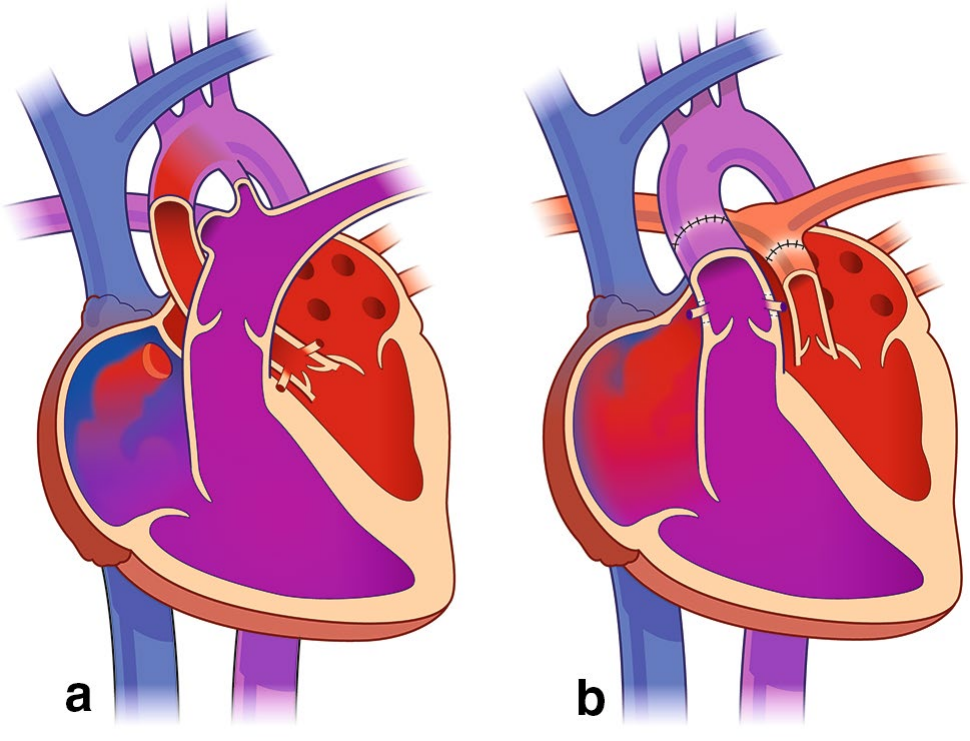
**a** Presents a diagram illustrating hypoplasia of the left ventricle [10]. The great vessels are normally related. The LV is not apex forming, but pulsatile. The aortic valve and mitral valve are hypoplastic but not atretic. There is an atrial septal defect and a patent ductus arteriosus. There is retrograde flow in the aortic arch. **b** Presents the arterial switch in the setting of LV hypoplasia and normally related great vessels. An extensive atrial septectomy is performed allowing for atrial level mixing and pulmonary venous inflow to the RA-RV-Aorta axis. The LV provides antegrade systolic pulmonary blood flow. The ductus arteriosus has been ligated and divided. The coronary arteries are translocated to the main PA root with coronary button defects filled with patch material in the native aortic root. A pulsatile small LV that could generate a systolic pressure of 25–40 mmHg is inadequate for the systemic output but may support pulmonary blood flow

A number of strategies have been developed to treat or palliate the newborn with “hypoplasia” of the non-apex forming LV, including the modified Norwood procedure, biventricular repairs and staged ventricular recruitment [7]. We have reported the use of stage 1 hybrid procedure in the setting of inadequate LV and severe aortic stenosis [8], followed LV rehabilitation, by takedown of the hybrid, Ross-Konno procedure, resection of endocardial fibroelastosis, and arch reconstruction. We now propose that the arterial switch operation may be an option for the newborn or small infant with hypoplasia of the LV where the LV is still pulsatile and can generate a pressure as low as 25 mmHg. By performing an arterial switch in this clinical scenario, the small but pulsatile LV can be incorporated in the pulmonary circulation and support pulmonary blood flow. The RV is used for systemic circulation using this approach. An extensive atrial septectomy would be required, and later bidirectional cavopulmonary anastomosis can be used for 1.5 ventricle repair (Fig. 8). The LV to PA connection, after arterial switch would act as a source of pulmonary blood flow, not dissimilar from an RV-PA shunt used in the Sano modification of the Norwood procedure, without the need for a right ventriculotomy. However, intracardiac streaming patterns may be of concern. The lack of ventriculotomy mitigates potential postoperative low cardiac output. Furthermore this approach may not require the use of systemic-to-pulmonary arterial shunting, hence avoiding a higher Qp:QS, low output syndrome, and an increased myocardial oxygen demand to supply ratio. There may not be a need for staging to Fontan, but Glenn could be required to enhance pulmonary blood flow at a later stage, and a hemi-Mustard [9], can be added later for complete septation with a pulsatile pulmonary circulation.

### Implications of the Model for Examining RV Failure

Heart failure is a significant cause of morbidity and mortality in patients with congenital heart disease, especially for the subset whose right ventricle functions as the systemic ventricle. The physiological and molecular differences in the right ventricle (RV) and left ventricle (LV) lead to different abilities to adapt to adverse conditions and respond to pharmacological therapeutics. Currently data describing the molecular changes that occur in the systemic RV are lacking. Available animal models create an RV with volume and/or pressure overload; however, the LV remains at high pressure. Ventricular-ventricular interactions have a critical impact on cardiac function and are likely to alter gene expression independent of RV function. A novel large animal model as reported herein creates a high pressure systemic RV with a low pressure subpulmonic LV by performing an arterial switch followed by an atrial septectomy to allow sufficient oxygenation via atrial mixing. To identify molecular regulatory networks and signaling pathways that are perturbed in the systemic RV, transcriptomic analysis can be performed to compare the gene expression profiles of the systemic RV and normal RV. One can then determine whether gene expression of the reloaded ventricle (post arterial switch RV) recapitulates a fetal RV gene program or whether new gene expression pathways are activated, potentially providing novel therapeutic targets.

### Future Directions

Possible modifications for this model include the use of strategies to further enhance atrial mixing and optimize midterm survival. The major issues with the current model are hypoxia and bleeding. We are considering incorporation of the Baffes’ procedure to the arterial switch. Baffes excised and transferred the right pulmonary veins to the right atrium and used an aortic homograft to join the IVC to the divided proximal end of the pulmonary veins [11]. This directs pulmonary venous blood to the right atrium and systemic venous blood to the left atrium, thus enhancing atrial mixing. Although we initially chose the pig model system for this project due to its close molecular homology to humans, similarity to the human heart in anatomy and conserved changes in RV systolic pressure following birth in piglets compared to human infants [12–14] we are also considering an ovine model given its more favorable cardiac anatomy and hemostasis noted in our prior experience with fetal and neonatal surgery in lambs [15–19].

## Conclusion

We report a porcine model of RV failure that is created with an arterial switch operation on standard anatomy great vessels. The model may have implications for studying the failing systemic RV and may support a novel approach for the management of borderline, pulsatile left ventricles.

## Data Availability

The data and material that support the findings of this study are available from the corresponding author, JPC, upon reasonable request.

## References

[1] Qamar ZA, Goldberg CS, Devaney EJ, Bove EL, Ohye RG (2007). Current risk factors and outcomes for the arterial switch operation. Ann Thorac Surg.

[2] Wernovsky G, Allen HD, Gutgesell HP, Clark EB, Driscoll DJ (2000). Transposition of the great arteries. Moss and Adams’ heart disease in infants, children, and adolescents: including the fetus and young.

[3] Duncan BW, Poirier NC, Mee RB, Graney JA, Malek CA, Latson LA (2004). Selective timing for the arterial switch operation. Ann Thorac Surg.

[4] Pretre R, Tamisier D, Bonhoeffer P, Mauriat P, Pouard P, Sidi D (2001). Results of the arterial switch operation in neonates with transposed great arteries. Lancet.

[5] Falkenberg C, Hallhagen S, Nilsson K, Nilsson B, Ostman-Smith I (2010). A study of the physiological consequences of sympathetic denervation of the heart caused by the arterial switch procedure. Cardiol Young.

[6] de la Riviere AB, Quaegebeur JM, Hennis PJ, de la Riviere GB, Huysmans HA, Brom AG (1983). Growth of an aorta-coronary anastomosis, an experimental study in pigs. J Thorac Cardiovasc Surg.

[7] Emani S, McElhinney DB, Tworetzky W, Myers PO, Schroeder B, Zurakowski D (2012). Staged left ventricular recruitment after single-ventricle palliation in patients with borderline left heart hypoplasia. J Am Coll Cardiol.

[8] Moon-Grady AJ, Moore P, Azakie A (2011). Ross-Konno and endocardial fibroelastosis resection after hybrid stage I palliation in infancy: Successful staged left-ventricular rehabilitation and conversion to biventricular circulation after fetal diagnosis of aortic stenosis. Pediatr Cardiol.

[9] Malhotra SP, Reddy VM, Qiu M, Pirolli TJ, Barboza L, Reinhartz O (2011). The hemi-mustard/bidirectional Glenn atrial switch procedure in the double-switch operation for congenitally corrected transposition of the great arteries: rationale and midterm results. J Thorac Cardiovasc Surg.

[10] Crucean A, Alqahtani A, Barron DJ, Brawn WJ, Richardson RV, O’Sullivan J (2017). Re-evaluation of hypoplastic left heart syndrome from a developmental and morphological perspective. Orphanet J Rare Dis.

[11] Mavroudis C, Backer CL, Siegel A, Gevitz M (2014). Revisiting the Baffes operation: its role in transposition of the great arteries. Ann Thorac Surg.

[12] Dixon JA, Spinale FG (2009). Large animal models of heart failure: a critical link in the translation of basic science to clinical practice. Circ Heart Fail.

[13] Wernersson R, Schierup MH, Jørgensen FG, Gorodkin J, Panitz F, Staerfeldt HH (2005). Pigs in sequence space: a 0.66X coverage pig genome survey based on shotgun sequencing. BMC Genom.

[14] Rudolph AM (1970). The changes in the circulation after birth. Their importance in congenital heart disease. Circulation.

[15] Azakie A, Carney JP, Lahti ML, Seiberlich MK, Hiremath G, Moklyak YO (2020). Feasibility study of catheter-based interventions for anisotropic expanded polytetrafluoroethylene cardiovascular conduits in a growing lamb model. J Invest Surg.

[16] Azakie A, Carney JP, Lahti ML, Mokylak Y, Bianco RW (2020). Anisotropic polytetrafluoroethylene cardiovascular conduits spontaneously expand in a growing lamb model. J Investig Surg.

[17] Reimer J, Syedain Z, Haynie B, Lahti M, Berry J, Tranquillo R (2016). Implantation of a tissue-engineered tubular heart valve in growing lambs. Ann Biomed Eng.

[18] Syedain Z, Reimer J, Lahti M, Berry J, Johnson S, Tranquillo RT (2016). Tissue engineering of acellular vascular grafts capable of somatic growth in young lambs. Nat Commun.

[19] Hofferberth SC, Saeed MY, Tomholt L, Fernandes MC, Payne CJ, Price K (2020). A geometrically adaptable heart valve replacement. Sci Transl Med.

